# Role of Artificial Intelligence in the Assessment of Postoperative Pain in the Pediatric Population: A Systematic Review

**DOI:** 10.7759/cureus.77074

**Published:** 2025-01-07

**Authors:** Ankit Kasundra, Roshan Chanchlani, Babu Lal, Suresh K Thanveeru, Geetesh Ratre, Reyaz Ahmad, Pramod K Sharma, Amit Agrawal

**Affiliations:** 1 Pediatric Surgery, All India Institute of Medical Sciences, Bhopal, IND; 2 Trauma and Emergency Medicine, All India Institute of Medical Sciences, Bhopal, IND; 3 Neurosurgery, All India Institute of Medical Sciences, Bhopal, IND

**Keywords:** artificial intelligence, deep learning, machine learning, pediatric, postoperative pain, post-surgical pain

## Abstract

Effective postoperative pain relief is crucial for the recovery of pediatric patients. While artificial intelligence (AI) is increasingly being applied in pain assessment, there is a notable lack of data regarding its role in managing postoperative pain in children. This systematic review aims to address this gap by focusing on AI’s use in predicting and evaluating pediatric postoperative pain. We conducted a comprehensive search of relevant studies from January 2000 to November 2023, identifying 4,491 studies, which were narrowed down to eight based on the Preferred Reporting Items for Systematic Reviews and Meta-Analyses guidelines. These selected studies included 4,470 pediatric patients assessed using various pain measurement tools. The AI models used, primarily deep learning and machine learning, demonstrated accuracy rates ranging from 79% to 85.62% and area under the receiver operating characteristic curve values between 84.00% and 94.00%. Although these AI-based pain assessment tools are still in the early stages, they often focus on single parameters. The heterogeneity of the available publications prevented the conduct of a meta-analysis. Our findings underscore the need for multimodal, multicentric research to improve the performance of AI-based tools for assessing postoperative pain in the pediatric population. Such advancements could significantly enhance the future of pediatric pain management.

## Introduction and background

Postoperative pain management in children presents a unique challenge, primarily due to communication barriers and the subjective nature of pain perception [1]. Effective pain relief requires accurate pain assessment, yet conventional methods commonly used in the pediatric population have notable limitations. Traditional pain assessment tools, which often rely on subjective measures and caregiver input, can lack reliability due to varying perceptions among individuals.

In contrast, innovative approaches utilizing artificial intelligence (AI) have shown promise in managing postoperative pain in adults. These strategies include analyzing facial expressions, body movements, and vocalizations to assess pain [2]. Other methods leverage natural language processing to evaluate verbal and written expressions related to pain [3]. Additionally, wearable devices equipped with sensors and intelligent monitoring capabilities provide real-time physiological data, such as heart rate, to help identify pain [4]. Integrating AI into decision support systems can assist healthcare providers in making more informed decisions regarding pain assessment and tailoring management strategies to meet individual patient needs [5-7].

Given the lack of consolidated data on AI tools for assessing postoperative pain in pediatric populations, our systematic review aims to explore the potential of AI in overcoming these challenges and enhancing postoperative pain evaluation in children. The goal is to offer an overview of the role of AI and its subfields in creating tools specifically designed to assess postoperative pain in pediatric populations.

## Review

Methods

This review followed the Preferred Reporting Items for Systematic Reviews and Meta-Analyses (PRISMA) guidelines [6]. The protocol was pre-registered in PROSPERO (CRD42023481401).

In this study, using AI for postoperative pain assessment in pediatric patients was analyzed through a systematic approach guided by the PICOST criteria. These criteria defined the scope and inclusion parameters for the review. The population included pediatric patients under the age of 18 years, ensuring that the focus remained on this specific demographic. The intervention examined was the application of AI and its subsets, highlighting the role of AI as a tool for evaluating postoperative pain. The study did not require a control group, as the focus was on assessing the performance of AI models rather than comparing them to alternative methods. The outcome measured was the effectiveness and accuracy of AI-based systems in assessing pain levels. The study design encompassed cross-sectional, retrospective, and prospective studies, reflecting a comprehensive evaluation of past and ongoing research. Finally, the timeframe included studies published between January 2000 and November 2023, ensuring the inclusion of the most relevant and up-to-date research in this field.

Certain exclusion criteria were applied to minimize potential biases and maintain methodological rigor. Studies involving adult populations, non-English articles, in vitro or animal experiments, reviews, editorials, book chapters, or studies with insufficient data were excluded. This ensured the results were tailored to pediatric patients and relied on robust, well-documented data. By applying these criteria, the study sought to systematically evaluate the role of AI in pediatric postoperative pain assessment, focusing on its accuracy, reliability, and potential for clinical application.

Search Strategy

A comprehensive search strategy was developed to identify relevant studies. Terms related to “postoperative pain” and synonyms such as “post-surgical pain”, “post-operative analgesia”, “pain assessment”, and “pain scale” were included in the search. Boolean operators were used to structure the query effectively: OR was applied to group synonyms, and AND was used to combine these groups with other key terms like “artificial intelligence”, “AI”, “machine learning”, and “pediatric patients”, or “children”. Parentheses were used to structure the search logic, for example: (“postoperative pain” OR “post-surgical pain” OR “pain assessment”) AND (“artificial intelligence” OR “machine learning”) AND (“pediatric” OR “children”).

The search was conducted in electronic databases such as PubMed, Scopus, and Web of Science. The filters were applied to limit results to studies published between January 2000 and November 2023 and written in English. By clearly outlining the terms, operators, and databases used, this strategy ensures replicability and enables other researchers to verify or expand upon the findings.

Data Extraction

Two reviewers independently analyzed the included studies. Screening was initially based on titles and abstracts, followed by a detailed review of shortlisted articles. Discrepancies were resolved through mutual consensus. Data extraction was performed using a standardized form. It included author, publication year, country, study design, patient demographics, pain scales, datasets, face detection software, AI algorithms, and performance metrics like accuracy and area under the receiver operating characteristic curve (AUC).

Risk of Bias

The risk of bias was assessed using the JBI critical appraisal tool. Two reviewers independently evaluated the study quality, and the results were finalized through discussion. Studies meeting over 70% of the criteria were classified as low risk, those meeting 50-70% as moderate risk, and studies below 50% as high risk.

Results

Article Selection

**Figure 1:**
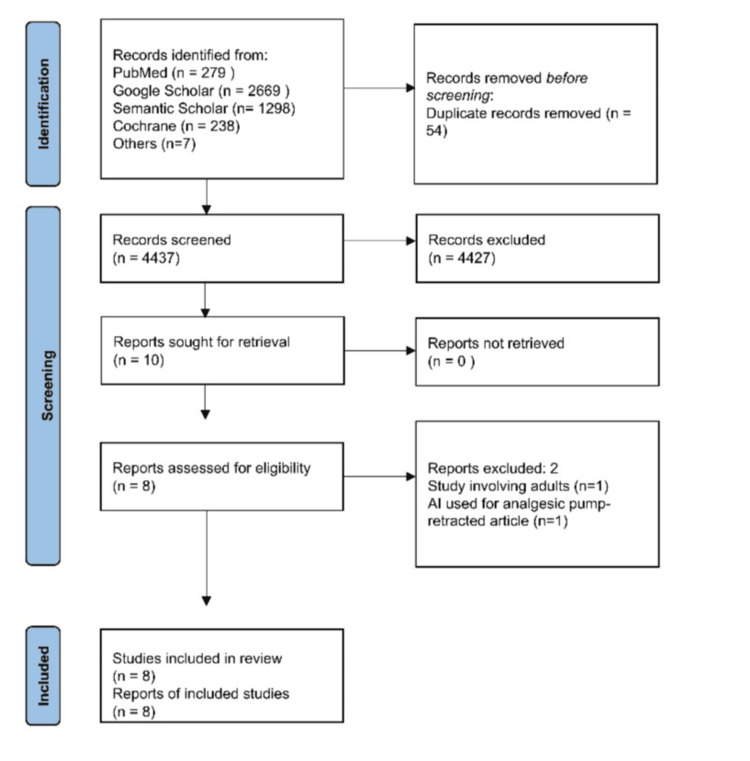
Identification of studies versus databases and registers

The search yielded 4,641 articles, with 55 duplicates removed. After screening 4,587 articles, nine met the eligibility criteria for full-text review. Two articles were excluded for reasons listed in Table 1, leaving seven studies for inclusion. The PRISMA flow diagram illustrates the selection process (Figure 1).

**Table 1:** Reasons for the studies excluded

Study	Reasons for exclusion
Fontaine et al., 2022 [8]	Study involving adult subjects
Zhang et al., 2022 [9]	AI used for analgesic pump postoperative retracted article

Study Characteristics

A total of eight studies, including 4,327 patients, were selected. Geographically, three studies were from Florida by the same author, one from China, one from Colombia, one from Turkey, and one from California. The earliest study was by Sikka et al. [10] in 2015, while the most recent were by Fang et al. [11] and Aydın and Özyazıcıoğlu [12] in 2023. Two studies used prospective designs; two were retrospective, and three were cross-sectional (Table 2).

**Table 2:** Characteristics of included studies FLACC: face, leg, activity, cry, and consolability; N-PASS: neonatal pain, agitation, and sedation scale; NRS: numerical rating scale; NA: not available; WBS: Wong-Baker faces pain scale; VAS: visual analog scale; CPEC: clinical pain expression of children; CERT: computer expression recognition toolbox; USF-MNPAD-I: University of South Florida multimodal neonatal pain assessment dataset (this includes video (face, head, and body), audio (crying sound), vital signs (heart rate, blood pressure, oxygen saturation), and cortical activity); DL: deep learning; ML: machine learning; LSTM: long short-term memory; RNN: recurrent current neural networks; CPANN: children pain assessment neural network; AUC: area under the receiver operating characteristic curve; ICC: intraclass correlation coefficient; HUSVF: high-utility surveillance and video face; CVML: computer vision and machine learning; NIPS: neonatal infant pain scale; MFCC: mel-frequency cepstral coefficients; FACET: facial action coding system engine technology

Study author (year)	Country	Study design	Age in years	Number of subjects (M/F)	Objective	Pain scores used	Dataset and tools	Face detection software	AI model used	AI model performance
Salekin et al., 2021 [2]	Florida	Retrospective study	Neonates	45	Develop an approach - multimodal spatiotemporal approach	NIPS, N-PASS	USF-MNPAD-1	YOLO - face and body movements, crying - MFCC	DL (VGG-16 (visual features) and VGG-net (RNN_LSTM-deep features)	Accuracy - 79.00%, AUC - 87.00%
Sikka et al., 2015 [10]	California	Prospective study	7-18	45 (24/21)	To develop and evaluate a CVML approach to measuring pain	NRS	NA	CERT	ML (computer vision)	AUC - 84.00% to 94.00%
Fang et al., 2023 [11]	China	Cross-sectional study	0-14	4104 (2580/1524)	To present the CPEC dataset	FLACC for <7 years, FPS-R for >7 years	CPEC	Dual shot face detector	DL (CPANN)	Accuracy - 82.10%, macro-F1 score - 73.90%
Aydın and Özyazıcıoğlu, 2023 [12]	Turkey	Prospective study	8-18	83 (58/25)	To evaluate the use of computer-aided facial expression analysis	WBS, VAS	NA	Open face	ML	WBS - ICC (95% CI) - 0.355, p-value - 0.006; VAS - ICC (95% CI) - 0.414, p-value - 0.001
Jiménez-Moreno et al., 2021 [13]	Colombia	Cross-sectional study	0-3	50 (39/11)	To present a classification approach of facial expression of child pain	FLACC	HUSVF	DLIB library	DL (AlexNet and VGG 16, 19, face)	Accuracy: AlexNet - 58.30% to 67.90%, VGG-16 - 17.10% to 93.70, VGG-19 - 34.4 % to 92.90%, VVG-FACE - 29.50% to 83.60%
Salekin et al., 2021 [14]	Florida	Cross-sectional study	Neonates	45	First multimodal neonatal pain dataset	NIPS, N-PASS	USF-MNPAD-1	NA	None	NA
Salekin et al., 2022 [15]	Florida	Retrospective study	Neonates	45	To present attentional generative models	NIPS, N-PASS	USF-MNPAD-1	NA	DL (multimodal-attentional generative network)	Accuracy - 82.02%, AUC - 90.60%
Xu et al., 2018 [16]	California	Prospective study	12	143(94/49)	Enhance children's pain level assessment using computer vision and transfer learning for more robust recognition across varied environments	NRS	NA	iMotions software interates Emotient’s FACET technology	ML (transfer learning)	AUC - 72%

Patient Demographics

The population ranged in age from preterm neonates to 18 years. Three studies by Salekin et al. focused exclusively on neonates. Of the 4,327 patients, 4,104 were from a study by Fang et al. [11] in China. The three studies by Salekin et al. used the same cohort of 45 patients, with only nine assessed for postoperative pain. The remaining studies involved 178 patients.

Pain Scores and Data Sources

Pain scores used in non-neonatal studies included the face, leg, activity, cry, and consolability (FLACC) scale (used in two studies for children under seven years), the faces pain scale-revised (FPS-R) (used in one study for children over seven years), the numerical rating scale (NRS), the Wong-Baker faces pain scale (WBS), and the visual analog scale (VAS). Data sources included clinical pain expression of children (CPEC) and high-utility surveillance and video face (HUSVF) datasets in studies by Fang et al. [11] and Jiménez-Moreno et al. [13], respectively. Sikka et al. [10] and Aydın and Özyazıcıoğlu [12] did not specify their data sources.

Face Detection Software and AI Models

Face detection software varied across studies. Fang et al. used a dual-shot face detector and DLIP library. Jiménez-Moreno et al. [13] used the computer expression recognition toolbox (CERT), while Aydın and Özyazıcıoğlu employed Open Face. AI models included children pain assessment neural network (CPANN), convolutional neural network (CNN), computer vision and machine learning (CVML), and machine learning (ML) V2. Face and body movement detection utilized YOLO software while crying sounds were analyzed using mel-frequency cepstral coefficients (MFCC). AI models included CNN-long short-term memory (LSTM) (reported in 2021) and the attention generative multimodal network (featured in 2022).

Model Performance Metrics

Model performance metrics varied. Fang et al. reported an accuracy of 82.10%, while Jiménez-Moreno et al. reported 85.62%. Sikka et al. [10] reported that AUC values ranged from 84.00% to 94.00%. Fang et al. [11] also presented a macro-F1 score of 73.90%, while Jiménez-Moreno et al. [13] evaluated AI models like AlexNet, VGG-16, and VGG-19. Aydın and Özyazıcıoğlu [12] reported WBS intraclass correlation coefficient (ICC) (95% CI) at 0.355 (p=0.006) and VAS ICC (95% CI) at -0.414 (p=0.001). The attention generative model demonstrated an accuracy of 82.02% and an AUC of 90.60% in 2023. The CNN-LSTM model reported 79.00% accuracy and an AUC of 87.00% in 2022.

Risk of Bias and Publication Bias

Using the JBI critical appraisal tool [17], all included studies were assessed as having a low risk of bias. The results are summarized in Table 3.

**Table 3:** JBI clinical appraisal tool N/A: not applicable Item 1: Were the criteria for inclusion in the sample clearly defined? Item 2: Were the study subjects and the setting described in detail? Item 3: Was the exposure measured in a valid and reliable way? Item 4: Were objective, standard criteria used for measurement of the condition? Item 5: Were confounding factors identified? Item 6: Were strategies to deal with confounding factors stated? Item 7: Were the outcomes measured in a valid and reliable way? Item 8: Was appropriate statistical analysis used? Item 9: Overall appraisal [17]

Study author (year)	Item 1	Item 2	Item 3	Item 4	Item 5	Item 6	Item 7	Item 8	Item 9
Salekin et al., 2021 [2]	Yes	Yes	Yes	Yes	No	No	Yes	Yes	Included
Sikka et al., 2015 [10]	Yes	Yes	Yes	Yes	No	No	Yes	Yes	Included
Fang et al., 2023 [11]	Yes	Yes	Yes	Yes	No	No	Yes	Yes	Included
Aydın and Özyazıcıoğlu, 2023 [12]	Yes	Yes	Yes	Yes	No	No	Yes	Yes	Included
Jimenez-Moreno, 2021 [13]	Yes	Yes	Yes	Yes	No	No	Yes	Yes	Included
Salekin et al., 2021 [14]	Yes	Yes	N/A	Yes	N/A	N/A	N/A	N/A	Excluded
Salekin et al., 2022 [15]	Yes	Yes	Yes	Yes	No	No	Yes	Yes	Included
Xu et al., 2018 [16]	Yes	Yes	Yes	Yes	No	No	Yes	Yes	Included

Discussion

Managing postoperative pain in children remains a significant clinical challenge due to the inherently subjective nature of pain and the need for age-specific assessment tools. Traditional pain assessment methods have been categorized into subjective, behavioral, and mixed scales, each with strengths and limitations. For children over seven years who can effectively communicate their pain levels, scales such as VAS, NRS, and FPS-R are widely used and remain reliable for guiding analgesic administration. However, these scales heavily rely on the child’s self-reporting ability, posing challenges for younger or less cooperative patients. While offering a solution for neonates and toddlers, behavioral scales are prone to inter-rater variability and require considerable time and expertise, particularly in busy clinical settings. Integrating physiological parameters into some scales, such as the COMFORT scale, provides a more objective approach but introduces additional complexity [1].

In this context, introducing AI as a tool for pain assessment offers a transformative opportunity. AI models, particularly those leveraging ML and deep learning (DL) techniques, have demonstrated promising potential in enhancing the accuracy and efficiency of pain evaluation. However, clinicians should be aware of the inherent challenges and limitations highlighted by the studies reviewed.

The findings from the seven included studies reveal varying levels of accuracy and reliability in AI-based models, raising essential questions about their applicability in real-world clinical settings. For instance, Fang et al. [10] reported an 82.1% accuracy for their CPANN model. Yet, its reliance on a dataset focused on IV access puncture pain limits its generalizability to broader postoperative contexts. Similarly, Jiménez-Moreno et al. [13] reported a high accuracy of 92.9% using a VGG-19 model but noted limitations due to the small dataset size, which impacted the generalizability and caused sensitivity issues. Some studies also reported AUC as a metric to provide deeper insights into the performance of these models. AUC measures the ability of a model to distinguish between different pain levels, with values closer to 1.0 indicating excellent discriminatory power. For example, Sikka et al. [10] reported AUC values of 0.84 and 0.91, demonstrating good to exceptional performance. However, discrepancies between AUC and other metrics, such as Cohen’s kappa values (0.36 and 0.61), highlight the need for a multifaceted approach to evaluating AI models, as high AUC values may not always correlate with clinical utility.

The innovative approaches demonstrated in some studies, such as Salekin et al.’s [2] multimodal spatiotemporal DL method, highlight the potential of integrating multiple data modalities to assess pain. However, the study’s limitations, including a small dataset and short monitoring duration, underline the importance of scaling these methods to ensure clinical relevance. Similarly, Aydın and Özyazıcıoğlu, who utilized a machine learning V2 model focusing on facial analysis, showed potential for non-invasive pain assessment. Yet, reliance on facial expressions alone may limit accuracy in diverse populations with varying facial features and pain responses. These findings emphasize the critical importance of developing AI models that consider varied and multimodal data inputs to improve reliability.

From a clinical perspective, while AI holds significant promise in advancing pain assessment, its current applications remain experimental. Clinicians must be cautious in interpreting AI-derived pain assessments, especially given the variability in study designs, outcome measures, and data sources. Nonetheless, the potential for AI to reduce subjectivity, improve efficiency, and enable real-time pain monitoring represents a significant step forward in pediatric postoperative care. For clinicians, these findings underscore the need for AI models to be adaptable to different age groups, pain types, and surgical contexts. Neonates, for example, require models that integrate behavioral and physiological parameters, such as heart rate, oxygen saturation, and body movements, as demonstrated in some studies [14]. Yet, only one study reviewed incorporated such a multimodal approach, reflecting a significant gap in the current research landscape. Moreover, the absence of randomized controlled trials comparing AI-based assessments with traditional pain scales limits the ability to draw robust conclusions about their efficacy. By addressing the limitations identified in this review, future research can help bridge the gap between experimental findings and clinical implementation, ultimately improving pain management outcomes for children.

Limitations of the Study

This review faced several limitations that should be acknowledged. A meta-analysis was not conducted due to significant heterogeneity among the included studies. This heterogeneity arose from variations in study design (e.g., retrospective, prospective, and cross-sectional studies), patient populations (age ranges, surgical types, and demographic variability), and outcome measurement methods. The AI models used across studies differed substantially, ranging from traditional ML to advanced DL techniques. Furthermore, the studies reported performance metrics using diverse statistical measures, such as accuracy, AUC, macro-F1 scores, and Cohen’s kappa, which were not directly comparable due to differences in dataset characteristics and evaluation methods. For example, some studies relied solely on facial expression analysis [10,13], while others included multimodal parameters [14]. These discrepancies made it methodologically inappropriate to pool results into a single meta-analysis, as such an approach could lead to misleading conclusions.

Another limitation was the small sample sizes in most studies, which limited the statistical power and generalizability of findings. For instance, Jiménez-Moreno et al. [13] included only 50 patients, while Salekin et al. [2] focused on a dataset of just nine neonates. Small datasets not only constrain the applicability of findings to broader populations but also increase the risk of overfitting or underfitting in AI models, as seen in several studies. Moreover, the reliance on single-parameter pain assessment methods in most studies further reduced the robustness of the findings, with only one study employing a multimodal approach.

Future Directions

To advance the field of AI in pediatric postoperative pain assessment, several research priorities emerge from this review. First, future studies should address the heterogeneity observed by developing standardized protocols for AI model evaluation. This includes using diverse and representative datasets that cover a range of surgical procedures, age groups, and pain types to improve the generalizability of AI tools. Incorporating multimodal approaches that combine behavioral and physiological parameters, as demonstrated in Salekin et al.’s [2] study, should be prioritized to enhance model robustness.

Randomized controlled trials comparing AI-based assessments with traditional pain scales are essential to validate AI’s effectiveness and determine its clinical applicability. These trials should include larger sample sizes to ensure sufficient statistical power and minimize biases. Furthermore, researchers should report standardized metrics, such as accuracy, AUC, and macro-F1 scores, with clear explanations of their significance, allowing clinicians and researchers to interpret and compare results effectively.

Given the complexity of AI performance metrics and the potential for biases in small datasets, collaboration with statistical specialists is recommended in future studies. This can ensure the appropriate design and analysis of AI-based studies, accounting for the nuances of ML and deep learning models. Additionally, future research should explore real-time AI tools in clinical settings, assessing their feasibility, reliability, and impact on clinical workflows. By addressing these limitations and focusing on these future directions, the field can move toward developing AI tools that are reliable, accurate, and applicable across diverse pediatric populations and clinical scenarios.

## Conclusions

The applicability of different pain assessment scales varies with age in pediatric patients: “behavioral” for neonates or toddlers, “mixed” for school-going age, and “subjective/numerical” for ages >7 years. Behavioral and mixed pain scales are based on multiple parameters. The AI-based pain assessment tools are still in their infancy and are mostly considered only a single parameter. Additionally, the performance of these models is suboptimal. Available publications have a huge heterogeneity, precluding a meta-analysis. We believe that the AI-based approach for pain assessment tools in the pediatric age group appears promising, and the gaps highlighted in our study will stimulate further multicentric research in that direction.

## References

[1] Zieliński J, Morawska-Kochman M, Zatoński T (2020). Pain assessment and management in children in the postoperative period: a review of the most commonly used postoperative pain assessment tools, new diagnostic methods and the latest guidelines for postoperative pain therapy in children. Adv Clin Exp Med.

[2] Salekin MS, Zamzmi G, Goldgof D, Kasturi R, Ho T, Sun Y (2021). Multimodal spatio-temporal deep learning approach for neonatal postoperative pain assessment. Comput Biol Med.

[3] Naseri H, Kafi K, Skamene S (2021). Development of a generalizable natural language processing pipeline to extract physician-reported pain from clinical reports: Generated using publicly-available datasets and tested on institutional clinical reports for cancer patients with bone metastases. J Biomed Inform.

[4] Avila FR, McLeod CJ, Huayllani MT (2021). Wearable electronic devices for chronic pain intensity assessment: a systematic review. Pain Pract.

[5] Pombo N, Araújo P, Viana J (2014). Knowledge discovery in clinical decision support systems for pain management: a systematic review. Artif Intell Med.

[6] Page MJ, McKenzie JE, Bossuyt PM (2021). The PRISMA 2020 statement: an updated guideline for reporting systematic reviews. BMJ.

[7] Aromataris E, Munn Z (2024). JBI manual for evidence synthesis.

[8] Fontaine D, Vielzeuf V, Genestier P (2022). Artificial intelligence to evaluate postoperative pain based on facial expression recognition. Eur J Pain.

[9] Zhang F, Wu S, Qu M, Zhou L (2022). Application of a remotely controlled artificial intelligence analgesic pump device in painless treatment of children. Contrast Media Mol Imaging.

[10] Sikka K, Ahmed AA, Diaz D, Goodwin MS, Craig KD, Bartlett MS, Huang JS (2015). Automated assessment of children’s postoperative pain using computer vision. Pediatrics.

[11] Fang J, Wu W, Liu J, Zhang S (2023). Deep learning-guided postoperative pain assessment in children. Pain.

[12] Aydın Aİ, Özyazıcıoğlu N (2023). Assessment of postoperative pain in children with computer assisted facial expression analysis. J Pediatr Nurs.

[13] Jiménez-Moreno C, Aristizábal-Nieto JK, Giraldo-Salazar OL (2021). Classification of facial expression of post-surgical pain in children: evaluation of convolutional neural networks. Visión Electrónica.

[14] Salekin MS, Zamzmi G, Hausmann J (2021). Multimodal neonatal procedural and postoperative pain assessment dataset. Data Brief.

[15] Salekin MS, Zamzmi G, Goldgof D (2022). Attentional generative multimodal network for neonatal postoperative pain estimation. Med Image Comput Comput Assist Interv.

[16] Xu X, Craig KD, Diaz D (2018). Automated pain detection in facial videos of children using human-assisted transfer learning. CEUR Workshop Proc.

[17] Moola S, Munn Z, Tufanaru C (2024). Systematic reviews of etiology and risk. JBI manual for evidence synthesis.

